# (*t*, *n*) Threshold *d*-Level Quantum Secret Sharing

**DOI:** 10.1038/s41598-017-06486-4

**Published:** 2017-07-25

**Authors:** Xiu-Li Song, Yan-Bing Liu, Hong-Yao Deng, Yong-Gang Xiao

**Affiliations:** 1 Chongqing University of Posts and Telecommunications, School of Computer Science and Technology, Chongqing, 400065 China; 2 Yangtze Normal University, College of Computer Engineering, Chongqing, 408000 China

## Abstract

Most of Quantum Secret Sharing(QSS) are (*n*, *n*) threshold 2-level schemes, in which the 2-level secret cannot be reconstructed until all *n* shares are collected. In this paper, we propose a (*t*, *n*) threshold *d*-level QSS scheme, in which the *d*-level secret can be reconstructed only if at least *t* shares are collected. Compared with (*n*, *n*) threshold 2-level QSS, the proposed QSS provides better universality, flexibility, and practicability. Moreover, in this scheme, any one of the participants does not know the other participants’ shares, even the trusted reconstructor *Bob*_1_ is no exception. The transformation of the particles includes some simple operations such as *d*-level CNOT, Quantum Fourier Transform(QFT), Inverse Quantum Fourier Transform(IQFT), and generalized Pauli operator. The transformed particles need not to be transmitted from one participant to another in the quantum channel. Security analysis shows that the proposed scheme can resist intercept-resend attack, entangle-measure attack, collusion attack, and forgery attack. Performance comparison shows that it has lower computation and communication costs than other similar schemes when 2 < *t* < *n* − 1.

## Introduction

A dealer who wants to share a secret among a group of participants, usually splits the secret into a few pieces. These pieces of the secret is called shares, which are distributed to different participants, and a share is only held by a participant. The secret can be reconstructed only when enough participants collaborate together. This is the basic idea of Secret Sharing (SS) in modern cryptography. A significant role of SS is that it protects secret information from being lost, destroyed, or altered. Therefore, SS is widely applied to threshold signature, threshold cryptography, secure multi-party computation, and group key management, etc.

Quantum Secret Sharing (QSS) is the expansion of SS in the quantum cryptography field, and the difference between the two is that QSS’ security is based on the fundamental principle of quantum physics. As a cryptographic scheme, QSS uses quantum information to deal with the problem of sharing classical or quantum secret. That is to say, the dealer distributes a secret that may be classical message or an unknown quantum state among a group of participants, and reconstructing the secret need a certain number of participants to collaborate together. The first QSS scheme was proposed by Hillery *et al*.^1^ in 1999, based on Greenberger-Home-Zeilinger(GHZ) state. Since then, many design and analysis schemes on QSS have been proposed^2–20^ such as circular QSSs^2–4^, dynamic QSSs^5, 6^, single particle QSSs^7–9^, graph state QSSs^10–12^, verifiable QSSs^13–15^, and other QSSs that may be based on Calderbank–Shor–Steane codes^16^, or based on phase shift operation^17–19^, or based on quantum search algorithm^20^.

According to different threshold, the existing QSS schemes can be classified into two categories: (*n*, *n*) QSS^2–12, 18–20^ and (*t*, *n*) QSS^10, 11, 13–17^. For the former, the secret cannot be reconstructed until all *n* shares are collected. For the latter, the secret can be reconstructed only if at least *t* shares are collected. Furthermore, these QSS schemes can be fallen into two categories: 2-level QSS^2–6, 10, 12, 17–20^ and *d*-level QSS^7–9, 11^ depending on the dimension of Hilbert space. For the former, the quantum secret and its shares are all in 2 dimension Hilbert space. For the latter, the dimension of the quantum states is more than 2, that is *d* > 2. In general, QSS uses different levels of authority to control the participants’ access privileges. Though each participant holds a share, only the qualified subsets of the participants can reconstruct the secret. All the qualified subsets are decided according to different application requirements. Each qualified subset may have different number of participants, and a participant may belong to several qualified subsets. To the (*t*, *n*) threshold QSS scheme, the number of participants of each qualified subset is *t*.

Compared with (*n*, *n*) QSS, the design of (*t*, *n*) QSS is more complex, because it need employ the technologies such as graph state or error-correcting encoding. In term of practice, (*t*, *n*) QSS is more flexible, because the reconstruction of a secret for (*t*, *n*) QSS need at least *t* participants whereas for (*n*, *n*) QSS must be *n* participants. Compared to 2-level QSS, the design of *d*-level QSS is more difficult. The main reason is that the operations of the quantum computational cell need higher dimensional unitary operations, such as quantum Fourier transform (QFT), *d*-dimensional Pauli operations, etc. In addition, the universality and practicability of *d*-level QSS are better than that of 2-level QSS, because the dimension of Hilbert space may be *d*, which is higher than 2.

Inspired by the flexibility of (*t*, *n*) threshold and the universality of *d*-level, in this paper, we propose a (*t*, *n*) threshold *d*-level QSS scheme. The scheme has generic properties of (*t*, *n*) threshold SS, e.g., the dealer Alice distributes *n* shares to *n* participants, and each participant only holds a share; any *t* out of the *n* participants can reconstruct the original secret. In addition, compared with the existing QSS schemes, the proposed QSS has better properties as follows. Owing to items 1 and 2, it provides lower computation cost; owing to item 3, it provides lower communication cost; owing to item 4, it is safer in resisting some common attacks.

- There only exist simple operations such as quantum Fourier transform (QFT) and generalized Pauli operator. The complex operations, e.g., the graph state or error-correcting encoding, do not appear in our scheme;
- Only the participant *Bob*_1_ need apply quantum Fourier transform (QFT) to his own particle, other participants do not need;
- It is unnecessary to transmit the quantum particles from one participant to the next in order;
- Any one of the participants does not know the other participants’ shares, even the trusted reconstructor *Bob*_1_ is no exception.

## Preliminaries

In this section, the related preliminaries are introduced including quantum Fourier transform (QFT) and inverse quantum Fourier transform (IQFT), generalized Pauli operator, and Shamir’s (*t*, *n*) threshold SS. These preliminaries will be used in presenting (*t*, *n*) threshold QSS scheme.

### Quantum Fourier Transform and Inverse Quantum Fourier Transform

#### **Definition 1**

. Quantum Fourier transform (QFT), a quantum version of the standard discrete Fourier transform, is a unitary transformation of *d*-level quantum system. For *y*, $x\in \{0,1,\ldots ,d-1\}$, the QFT is defined by refs 21 and 22

1 $$QFT|y\rangle =\frac{1}{\sqrt{d}}\sum _{x=0}^{d-1}\,{\omega }^{y\cdot x}|x\rangle ,$$

where $\omega ={e}^{2\pi i/d}$ is a primitive *d*-th root of unity.

#### **Definition 2**

. For *x*, $y\in \{0,1,\ldots ,d-1\}$, the inverse quantum Fourier transform (IQFT) is defined by

2 $$QF{T}^{-1}|x\rangle =\frac{1}{\sqrt{d}}\sum _{y=0}^{d-1}\,{\omega }^{-x\cdot y}|y\rangle .$$

Between the QFT and the IQFT, there exists the relationship given by

3 $$QF{T}^{-1}(QFT|y\rangle )=QF{T}^{-1}(\frac{1}{\sqrt{d}}\sum _{x=0}^{d-1}\,{\omega }^{y\cdot x}|x\rangle )=|y\rangle .$$

### Pauli Operator

#### **Definition 3**

. On Hilbert space of *d*-level quantum system, the generalized Pauli operator is defined by ref. 23

4 $${U}_{\alpha ,\beta }=\sum _{x=0}^{d-1}\,{\omega }^{\beta \cdot x}|x+\alpha \rangle \langle x|,$$

where *α*, $$\beta \in \{0,1,\ldots ,d-1\}$$.

In particular, on Hilbert space of *d*-level quantum system, the *X* gate and *Z* gate are represented by ref. 24

5 $$X={U}_{1,0}=\sum _{x=0}^{d-1}\,|x+1\rangle \langle x|,\quad Z={U}_{0,1}=\sum _{x=0}^{d-1}\,{\omega }^{x}|x\rangle \langle x|.$$

### Shamir’s (*t*, *n*) threshold SS

#### **Definition 4**

. Suppose that there are a trusted dealer and *n* participants $P=\{{P}_{1},{P}_{2},\ldots ,{P}_{n}\}$, Shamir’s (*t*, *n*) threshold SS^25^ consists of the following two algorithm:

Share generation algorithm: The dealer randomly chooses a polynomial with degree *t* − 1: $f(x)={a}_{0}+{a}_{1}x+{a}_{2}{x}^{2}+\cdots +{a}_{t-1}{x}^{t-1}$, where $({a}_{0},{a}_{1},\ldots ,{a}_{t-1})\in {Z}_{d}^{t}$, and *a*_0_ is a secret. The dealer computes *n* shares *f*(*x*_*i*_) for $(i=1,2,\ldots ,n)$, then he/she sends *n* shares to *n* participants via a secure channel, and each participant *P*_*i*_ holds only a share *f*(*x*_*i*_).

Secret reconstruction algorithm: There are *n* distinct points $\{({x}_{i},f({x}_{i}))|i=1,2,\ldots ,n\}$ on the polynomial *f*(*x*) in the 2-dimensional plane, so if and only if at least *t* points $\{({x}_{r},f({x}_{r}))|r=1,2,\ldots ,t\}$ are given, the polynomial *f*(*x*) can be reconstructed by using the Lagrange interpolation formula as follows

6 $$f(x)=\sum _{r=1}^{t}\,f({x}_{r})\prod _{1\le j\le t,j\ne r}\,\frac{x-{x}_{j}}{{x}_{j}-{x}_{r}}.$$

If any *t* out of the *n* participants, denoted by $R=\{{P}_{1},{P}_{2},\ldots ,{P}_{t}\}$, take out their shares $({x}_{r},(f({x}_{r})))\,for\,$ $(r=1,2,\ldots ,t)$. Then the *t* participants can reconstruct the original secret *a*_0_ based on the above Equation (6)

7 $${a}_{0}=f\mathrm{(0)}=\sum _{r=1}^{t}\,f({x}_{r})\prod _{1\le j\le t,j\ne r}\,\frac{{x}_{j}}{{x}_{j}-{x}_{r}}.$$

## Results

### The Proposed QSS Scheme

Suppose that Alice is a dealer, and $B=\{Bo{b}_{1},Bo{b}_{2},\ldots ,Bo{b}_{n}\}$ is a set of *n* participants. Alice chooses any one of the participants *Bob*_1_ as a trusted reconstructor. The role of *Bob*_1_ is to collect any *t* shares from *n* participants and reconstruct the final secret. The proposed QSS scheme consists of three phases: initialization phase, share distribution phase, and secret reconstruction phase.

#### Initialization Phase

Alice first finds a suitable prime *d* satisfying $n\le d\le 2n$ and sets a finite field *Z*_*d*_. To divide a secret ${a}_{0}\in {Z}_{d}$ into *n* pieces, Alice randomly picks a polynomial with degree *t* − 1: $f(x)={a}_{0}+{a}_{1}x+{a}_{2}{x}^{2}+\cdots$ $+{a}_{t-1}{x}^{t-1}$, where the coefficients $a=({a}_{1},\ldots ,{a}_{t-1})\in {Z}_{d}^{t-1}$ are randomly chosen, and the symbol + means addition modulo *d*.

#### Share Distribution Phase

Similar to Share generation algorithm of Shamir’s (*t*, *n*) threshold SS, Alice selects *n* distinct and nonzero values ${x}_{i}\in {Z}_{d}$ to compute *n* shares $f({x}_{i})\in {Z}_{d}$ for $(i=1,2,\ldots ,n)$, and then she publishes all *x*_*i*_. Each classical share *f*(*x*_*i*_) can be encoded in a random qubit string according to the encoding method of BB84 protocol^26^ or other secure quantum key distribution (QKD) protocols. After having finished the encoding procedure, Alice distributes sequentially the qubit string of *f*(*x*_*i*_) to the corresponding participant *Bob*_*i*_ for $(i=1,2,\ldots ,n)$ via a secure quantum channel. That is to say, each participant *Bob*_*i*_ holds a share *f*(*x*_*i*_). After having finished the distribution procedure of the qubit strings of all shares, the secret *a*_0_ is shared among a group of participants. In addition, Alice selects a Hash function $H()$ such as *SHA*1 to compute hash value *H*(*a*_0_), and sends it to the participant *Bob*_1_.

#### Secret Reconstruction Phase

**Figure 1:**
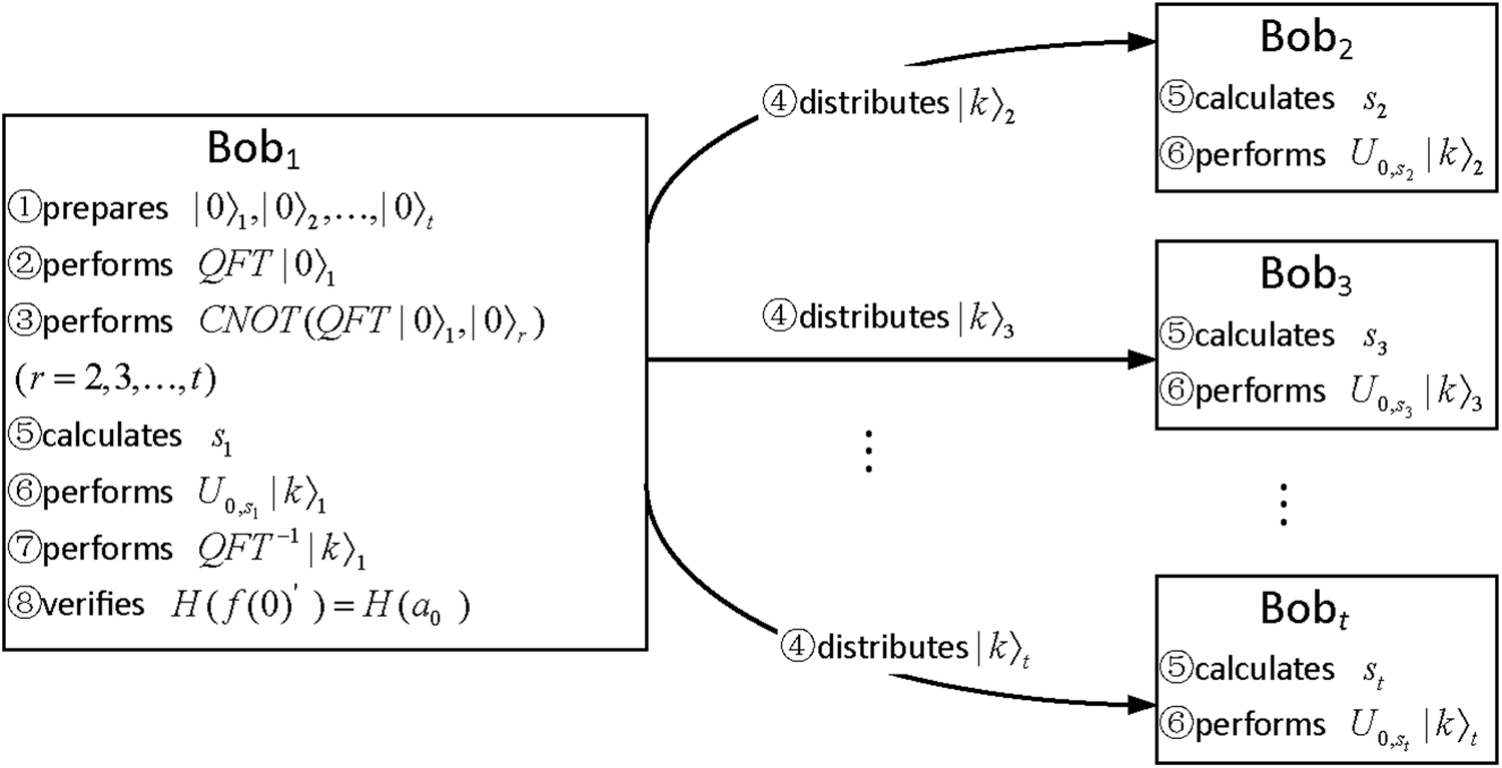
Reconstruction process of the original secret.

we assume that all qualified subsets of the participants are decided according to the specific application scenario, and the number of participants of each qualified subset is *t*. On a certain day, the secret *a*_0_ need to be reconstructed, any one of all qualified subsets is selected due to the absence of some participants. For simplicity of description, we assume that the selected qualified subset is denoted by $R=\{Bo{b}_{1},Bo{b}_{2},\ldots ,Bo{b}_{t}\}$. Figure 1 shows the reconstruction process of the original secret. In the process, each participant *Bob*_*r*_ $(r=2,3,\ldots ,t)$ performs the steps 5 and 6, and *Bob*_1_ performs the steps 1–8. The details of the reconstruction process are described as follows.

Step 1. As a trusted participant, *Bob*_1_ prepares *t* qudit particles ${|0\rangle }_{1},{|0\rangle }_{2},\ldots ,{|0\rangle }_{t}$, and each particle has *m* qubit, where $m=\lceil {{\rm{l}}{\rm{o}}{\rm{g}}}_{2}\,d\rceil$.

Step 2. Let $|0\rangle ,|1\rangle ,\ldots ,|d-1\rangle$ be a standard orthonormal basis of a *d*-level quantum system and set a *QFT* based on this orthonormal basis. When *Bob*_1_ applies the *QFT* to the first particle |0〉_1_, the composite state $|{\phi }_{1}\rangle$ of *t* particles is denoted by

8 $$\begin{array}{rcl}|{\phi }_{1}\rangle  & = & (QFT{|0\rangle }_{1})\,{|0\rangle }_{2}{|0\rangle }_{3}\cdots {|0\rangle }_{t}\\  & = & (\frac{1}{\sqrt{d}}\sum _{k=0}^{d-1}\,{\omega }^{0\cdot k}{|k\rangle }_{1})\,{|0\rangle }_{2}{|0\rangle }_{3}\cdots {|0\rangle }_{t}\\  & = & (\frac{1}{\sqrt{d}}\sum _{k=0}^{d-1}\,{|k\rangle }_{1})\,{|0\rangle }_{2}{|0\rangle }_{3}\cdots {|0\rangle }_{t},\end{array}$$

where $\omega ={e}^{2\pi i/d}$ is a primitive *d*-th root of unity.

Step 3. *Bob*_1_ performs respectively *d*-level *CNOT* operation on the particle |0〉_*r*_ for $(r=2,3,\ldots ,t)$. Where (*QFT* |0〉_1_) is the control qudit and |0〉_*r*_ is the target qudit. After performed (*t* − 1) *CNOT* operations by *Bob*_1_, the state $|{\phi }_{1}\rangle$ evolves as an entangled state

9 $$\begin{array}{rcl}|{\phi }_{2}\rangle  & = & (CNOT((QFT{|0\rangle }_{1}),{|0\rangle }_{2}))\otimes (CNOT((QFT{|0\rangle }_{1}),{|0\rangle }_{3}))\otimes \cdots \otimes (CNOT((QFT{|0\rangle }_{1},{|0\rangle }_{t})))\\  & = & \frac{1}{\sqrt{d}}\sum _{k=0}^{d-1}\,{|k\rangle }_{1}{|k\rangle }_{2}{|k\rangle }_{3}\cdots {|k\rangle }_{t}.\end{array}$$

Step 4. *Bob*_1_ sends respectively the particle |*k*〉_*r*_ $(r=2,3,\ldots ,t)$ to the corresponding participant *Bob*_*r*_ through the authenticated quantum channel.

Step 5. After all participants have received their particles, each participant *Bob*_*r*_ $(r=1,2,\ldots ,t)$ takes out his share *f*(*x*_*r*_) and calculates respectively the following value

10 $${s}_{r}=f({x}_{r})\prod _{1\le j\le t,j\ne r}\,\frac{{x}_{j}}{{x}_{j}-{x}_{r}}\,mod\,d.$$

For convenience, the *s*_*r*_ is named shadow of the share *f*(*x*_*r*_).

Step 6. Each participant *Bob*_*r*_ $(r=1,2,\ldots ,t)$ performs a generalized Pauli operator ${U}_{0,{s}_{r}}$ on his particle |*k*〉_*r*_, where ${U}_{0,{s}_{r}}$ is defined by

11 $${U}_{\mathrm{0,}{s}_{r}}=\sum _{k=0}^{d-1}\,{\omega }^{{s}_{r}\cdot k}{|k\rangle }_{rr}\langle k|.$$

After the Pauli operator ${U}_{0,{s}_{r}}$ $(r=1,2,\ldots ,t)$ is performed on each particle, the state $|{\phi }_{2}\rangle$ evolves as

12 $$\begin{array}{ccc}|{\phi }_{3}\rangle  & = & \frac{1}{\sqrt{d}}{\sum }_{k=0}^{d-1}\,{\omega }^{{s}_{1}\cdot k}{|k\rangle }_{1}{\omega }^{{s}_{2}\cdot k}{|k\rangle }_{2}{\omega }^{{s}_{3}\cdot k}{|k\rangle }_{3}\cdots {\omega }^{{s}_{t}\cdot k}{|k\rangle }_{t}\\  & = & \frac{1}{\sqrt{d}}{\sum }_{k=0}^{d-1}\,{\omega }^{({\sum }_{r=1}^{t}{s}_{r})\cdot k}{|k\rangle }_{1}{|k\rangle }_{2}{|k\rangle }_{3}\cdots {|k\rangle }_{t}\end{array}.$$

Step 7. *Bob*_1_ applies *QFT* ^−1^ to his own particle |*k*〉_1_ and further measures it in the computational basis to obtain the secret $f\mathrm{(0)}^{\prime} ={\sum }_{r=1}^{t}\,{s}_{r}\,mod\,d$.

Step 8. *Bob*_1_ first computes the hash value *H*(*f*(0)′) using a hash function $H()$, and then verifies $H(f\mathrm{(0)}^{\prime} )=H({a}_{0})$. If the equation holds, he shares the secret with other participants; otherwise he thinks that there is at least one dishonest participant and ends the reconstruction phase.

### Correctness Proof

The proposed (*t*, *n*) threshold QSS is proven in this section. The proof of correctness will focus primarily on Equation (12) of Step 6 and the secret recovery of Step 7.

#### **Lemma 1**

. If the Pauli operator ${U}_{0,{s}_{r}}={\sum }_{k=0}^{d-1}\,{\omega }^{{s}_{r}\cdot k}{|k\rangle }_{rr}\langle k|$ is performed on the particle |*k*〉_*r*_ $(r=1,2,\ldots ,t)$ of the orthogonal entangled state $|{\phi }_{2}\rangle$ of Equation (9) by the participant *Bob*_*r*_ $(r=1,2,\ldots ,t)$, the state $|{\phi }_{2}\rangle$ evolves as $|{\phi }_{3}\rangle$ of Equation (12).

#### **Proof**

. When the Pauli operator ${U}_{\mathrm{0,}{s}_{r}}={\sum }_{k=0}^{d-1}\,{\omega }^{{s}_{r}\cdot k}{|k\rangle }_{rr}\langle k|$ is performed on the particle |*k*〉_*r*_ of the state $|{\phi }_{2}\rangle$ of Equation (9) for $(r=1,2,\ldots ,t)$, the state $|{\phi }_{2}\rangle$ evolves as

13 $$\begin{array}{ccc}|{\phi }_{3}\rangle  & = & \frac{1}{\sqrt{d}}{\sum }_{k=0}^{d-1}\,{U}_{0,{s}_{1}}{|k\rangle }_{1}\otimes {U}_{0,{s}_{2}}{|k\rangle }_{2}\otimes \cdots \otimes {U}_{0,{s}_{r}}{|k\rangle }_{t}\\  & = & \frac{1}{\sqrt{d}}{\sum }_{k=0}^{d-1}\,{\omega }^{{s}_{1}\cdot k}{|k\rangle }_{11}{\langle k|k\rangle }_{1}{\omega }^{{s}_{2}\cdot k}{|k\rangle }_{22}{\langle k|k\rangle }_{2}\cdots {\omega }^{{s}_{t}\cdot k}{|k\rangle }_{tt}{\langle k|k\rangle }_{t}\\  & = & \frac{1}{\sqrt{d}}{\sum }_{k=0}^{d-1}\,{\omega }^{{s}_{1}\cdot k}{|k\rangle }_{1}{\omega }^{{s}_{2}\cdot k}{|k\rangle }_{2}\cdots {\omega }^{{s}_{t}\cdot k}{|k\rangle }_{t}\\  & = & \frac{1}{\sqrt{d}}{\sum }_{k=0}^{d-1}\,{\omega }^{({s}_{1}+{s}_{2}+\cdots +{s}_{t})\cdot k}{|k\rangle }_{1}{|k\rangle }_{2}\cdots {|k\rangle }_{t}\\  & = & \frac{1}{\sqrt{d}}{\sum }_{k=0}^{d-1}\,{\omega }^{({\sum }_{r=1}^{t}{s}_{r})\cdot k}{|k\rangle }_{1}{|k\rangle }_{2}\cdots {|k\rangle }_{t}.\end{array}\,$$

#### **Lemma 2**

. If *QFT* ^−1^ is applied to the particle |*k*〉_1_ of the state $|{\phi }_{3}\rangle$ of Equation (12), the measurement output of the transformed particle is the original secret $f\mathrm{(0)}={\sum }_{r=1}^{t}\,{s}_{r}\,mod\,d$.

#### **Proof**

. Based on Equation (10) and Lagrange interpolation formula of Equation (7), *f*(0) can be calculated by

14 $$\begin{array}{rcl}f\mathrm{(0)} & = & (f({x}_{1}){\prod }_{1\le j\le t,j\ne 1}\,\frac{{x}_{j}}{{x}_{j}-{x}_{1}}+f({x}_{2}){\prod }_{1\le j\le t,j\ne 2}\,\frac{{x}_{j}}{{x}_{j}-{x}_{2}}+\cdots \\  &  & +\,f({x}_{t}){\prod }_{1\le j\le t,j\ne t}\,\frac{{x}_{j}}{{x}_{j}-{x}_{t}})\,mod\,d\\  & = & ({s}_{1}+{s}_{2}+\cdots +{s}_{t})\,mod\,d\\  & = & ({\sum }_{r=1}^{t}{s}_{r})\,mod\,d.\end{array}$$

According to the Equation (3), *Bob*_1_ applies *QFT* ^−1^ to the first particle of the state $|{\phi }_{3}\rangle$ of Equation (12) and obtains

15 $$\begin{array}{c}QF{T}^{-1}(\frac{1}{\sqrt{d}}{\sum }_{k=0}^{d-1}\,{\omega }^{({\sum }_{r=1}^{t}{s}_{r})\cdot k}{|k\rangle }_{1})\\ \begin{array}{ccc} & = & |{\sum }_{r=1}^{t}\,{s}_{r}\,mod\,d\rangle \\  & = & |f(0)\rangle .\end{array}\end{array}\,$$

When *Bob*_1_ further measures the first particle in his hand, the measurement output is original secret *f*(0).

### Security Analysis

In this section, the security of the proposed (*t*, *n*) threshold QSS scheme is analyzed. The security analysis focuses primarily on intercept-resend attack, entangle-measure attack, collusion attack, and forgery attack.

#### Intercept-Resend Attack

Without loss of generality, Eve is assumed as an eavesdropper, who has unlimited computing power whose technology is only limited by the laws of quantum mechanics. Suppose Eve controls the quantum channel and intercepts any one quantum particle on the way from *Bob*_1_ to *Bob*_*r*_ $(r\in \{2,3,\ldots ,t\})$ in Step 4, then she measures the intercepted particle by using the computational basis $\{|0\rangle ,|1\rangle ,\ldots ,|d-1\rangle \}$. With the probability of 1/*d* she can succeed with the attack and get $k(k\in \{0,1,\ldots ,d-1\})$. Further she prepares a new particle that is the same as the intercepted one, and then resends the new particle to *Bob*_*r*_ $(r\in \{2,3,\ldots ,t\})$. Unfortunately, the measurement outcome *k* does not contain any information about private share *f*(*x*_*r*_) or its shadow *s*_*r*_. Therefore, Eve cannot get any valuable information in the intercept-resend attack.

#### Entangle-Measure Attack

In entangle-measure attack, the eavesdropper Eve may use a unitary operation to entangle an ancillary particle on the intercepted one, and then measures the ancillary particle to obtain valuable information. Suppose Eve intercepts all *t* − 1 particles transmitted from *Bob*_1_ to *Bob*_*r*_ $(r\in \{2,3,\ldots ,t\})$, and then prepares an ancillary particle |*e*〉_*a*_ $(e\in \mathrm{\{0,1,}\ldots ,d-\mathrm{1\}))}$. Further, she entangles the ancillary particle |*e*〉_*a*_ on any one of the intercepted particles such as |*k*〉_*u*_ by using *d*-level *CNOT* operation, where |*k*〉_*u*_ is the control qudit and |*e*〉_*a*_ is the target qudit. The state $|{\phi }_{2}\rangle$ of Equation (9) evolves as $|{\phi }_{2}\rangle$′

16 $$\begin{array}{ccc}|{\phi }_{2}{\rangle }^{{\prime} } & = & (CNOT({|k\rangle }_{u},{|e\rangle }_{a}))\,|{\phi }_{2}\rangle \\  & = & \frac{1}{\sqrt{d}}{\sum }_{k=0}^{d-1}\,{|k\rangle }_{1}{|k\rangle }_{2}\cdots {|k\rangle }_{u}\cdots {|k\rangle }_{t}{|k\oplus e\rangle }_{a}\end{array}.$$

Next step, Eve chooses another particle |*k*〉_*v*_ as control particle to perform *d*-level *CNOT* operation on the target particle |*e*〉_*a*_. Now the state $|{\phi }_{2}\rangle ^{\prime}$ evolves as $|{\phi }_{2}\rangle ^{\prime\prime}$

17 $$\begin{array}{ccc}|{\phi }_{2}{\rangle }^{\prime\prime} & = & (CNOT({|k\rangle }_{v},{|k\oplus e\rangle }_{a}))\,|{\phi }_{2}{\rangle }^{{\prime} }\\  & = & \frac{1}{\sqrt{d}}{\sum }_{k=0}^{d-1}\,({|k\rangle }_{1}{|k\rangle }_{2}\cdots {|k\rangle }_{v}\cdots {|k\rangle }_{t}{|k\oplus k\oplus e\rangle }_{a}\\  & = & |{\phi }_{2}\rangle {|e\rangle }_{a}\end{array}.$$

It can be seen that the ancillary particle |*e*〉_*a*_ is disentangled out from the entangled state $|{\phi }_{2}\rangle ^{\prime}$, and the original state $|{\phi }_{2}\rangle$ is not changed. If Eve measures the ancillary particle |*e*〉_*a*_, she obtains *e*, which is the same as prepared at the beginning. From this, Eve can come to the conclusion that the particles |*k*〉_*u*_ and |*k*〉_*v*_ are the same.

Suppose Eve takes each intercepted particle |*k*〉_*r*_ $(r=2,3,\ldots ,t)$ as control particle respectively, and |*e*〉_*a*_ as target particle to perform *d*-level *CNOT* operation. As a result, she finds all particles ${|k\rangle }_{2},{|k\rangle }_{3},\ldots ,{|k\rangle }_{t}$ are the same. Similar to the entangle-measure attack, the measurement outcome of the particle |*k*〉_*r*_ $(r=2,3,\ldots ,t)$ does not contain any information about private share *f*(*x*_*r*_) or its shadow *s*_*r*_. Therefore, Eve cannot also get any valuable information in the entangle-measure attack, only knowing that all transmitted particles ${|k\rangle }_{2},{|k\rangle }_{3},\ldots ,{|k\rangle }_{t}$ are the same.

#### Collusion Attack

As is known to all, QSS scheme uses the qualified subsets to prevent collusion attack of the participants. After analyzing the existing QSS schemes, we find some schemes cannot resist collusion attack, in which some participants can collude to get the private information of other participants. That is to say, in these QSS schemes, by getting rid of several qualified participants, the unqualified subsets of participants can reconstruct the original secret. Classifying collusion attacks of the existing QSS schemes, the study focuses on the following cases.

**Case 1**: Collusion attack of *Bob*_*e*−1_ and *Bob*_*e*+1_

In refs 17, 22 and 27, if *Bob*_*e*−1_ and *Bob*_*e*+1_ are dishonest, they can collude to get the private information of *Bob*_*e*_. The reason is that the refs 17, 22 and 27 have the same security loopholes: the private information of the previous participant is transformed by using the unitary operation, and then it is transmitted to the next participant. If *Bob*_*e*−1_ and *Bob*_*e*+1_ collaborate, *Bob*_*e*−1_ may send the particle transformed by himself such as ${U}_{e-1}\,{|k\rangle }_{e-2}$ to *Bob*_*e*+1_. As a result, *Bob*_*e*+1_ not only holds the particle ${U}_{e-1}\,{|k\rangle }_{e-2}$ transmitted by *Bob*_*e*−1_, but also holds the particle ${U}_{e}{U}_{e-1}\,{|k\rangle }_{e-2}$ transmitted by *Bob*_*e*_. Given this, *Bob*_*e*+1_ can calculate out *U*_*e*_ operation of *Bob*_*e*_, and further he can deduce the private information of *Bob*_*e*_.

**Case 2**: Collusion attack of the first participant *Bob*_1_ and the last participant *Bob*_*n*_

As ref. 6 pointed out there exists a security loophole in the dynamic QSS of ref. 5, i.e., the first participant and the last one can collude to obtain the master key of the dealer without the other participants’ cooperation. Ref. 4 also found that the circular QSS of ref. 3 is not secure as the first participant and the last one can illegally obtain the secret messages without introducing any error. The refs 3 and 5 also have the same security loopholes: the dealer and *n* participants transmit the transformed private information one by one. The transmission route forms a circle, in which the first participant is at the left of the dealer, and the last one is at the right of the dealer. If the first participant colludes with the last one, they can obtain the dealer’s private information.

Case 1 never happens in the proposed (*t*, *n*) threshold QSS scheme, because each participant performs unitary operation with private information in his own lab, and the transformed private information is not transmitted via the quantum channel. Case 2 never also happens in the proposed (*t*, *n*) threshold QSS scheme, because the dealer (*Alice*) and reconstructor (*Bob*_1_) do not take part in the circular transmission route, and their private information are not passed from one participant to the next but saved in their own hands. Therefore, as long as the dealer (*Alice*) and the reconstructor (*Bob*_1_) are both trusted entities, the proposed QSS scheme can resist collusion attack.

#### Forgery Attack

For secret sharing scheme, as always, it is an issue of public concern to prevent the participants from providing fake shares or shadows. In SS, Feldman^28^ first studied this problem and proposed a verifiable secret sharing, in which each participant’s share can be verified publicly. In QSS, Yang *et al*.^13, 14^ proposed two verifiable schemes to check whether some dishonest participants provide fake shares. Song *et al*.^15^ pointed out the forged quantum particles can pass the verification of other participants in ref. 13 and further proposed an new verifiable QSS scheme to improve the original one. From here we can see that verifiable QSS must provide validation function to resist forgery attack of the participants.

In the proposed QSS scheme, in order to resist forgery attack, the reconstructor *Bob*_1_ uses hash function $H()$ to certify the authenticity of the secret. During the secret reconstruction phase, if a dishonest participant *Bob*_*e*_ $(e\in \{2,3,\ldots ,t\})$ performs Pauli operator ${U}_{\mathrm{0,}{s}_{e^{\prime} }}$ with a fake shadow *s*_*e*′_ instead of his true *s*_*e*_, though other participants provide the correct information, the original secret *a*_0_ cannot be recovered correctly. In Step 8 of the secret reconstruction phase, when *Bob*_1_ calculates out the secret *f*(0)′ and verifies it by checking the equation $H(f\mathrm{(0)}^{\prime} )=H({a}_{0})$, he finds that the equation does not hold. He thinks that at least one dishonest participant has provided a fake shadow, and he terminates the reconstruction process and does not share the wrong secret *f*(0)′ with other participants. Therefore, the forgery attack of the participant *Bob*_*e*_ is infeasible.

### Performance Analysis and Comparison

In this section, the performance of the proposed QSS scheme is analyzed and compared with five other similar schemes: Yang *et al*.’s QSS of ref. 7, Qin *et al*.’s QSS of ref. 17, Shi *et al*.’s protocol I and protocol III of ref. 22, and Li *et al*.’s QSS of ref. 27. The performance analysis and comparison of the six similar schemes can be viewed from the following three aspects: universality and practicability, computation cost, and communication cost.

#### Universality and Practicability

In ref. 7, Yang *et al*. prepares an *n*-particle entangled state to design their protocol, and each participant holds a *d*-level particle. In ref. 17, the dealer prepares a multi-particle sequence, in which each particle is 2-level. In the protocol I and III of ref. 22, the initiator who is taken as one of the participants prepares a *d*-level 2-particle entangled state, and each of other *n* − 1 participants prepares respectively a *d*-level single particle. In ref. 27, the dealer prepares an ordered sequence of multiple EPR pairs. In the proposed QSS, the participant *Bob*_1_ prepares a *t*-particle entangled state by using *d*-level *CNOT* operation, and each participant holds a *d*-level particle.

We assume that the number of the prepared single particles or EPR pairs is the same as that of the participants who reconstruct the secret in the six similar schemes. In ref. 7 and the proposed scheme, each participant holds a particle, and each particle has *m* qubits, where $m=\lceil {\mathrm{log}}_{2}\,d\rceil$. As Table 1 shows, ref. 7 need prepare *mn* qubits, and the proposed QSS need prepare *mt* qubits. In the protocol I of ref. 22, the total number of the prepared particles is *n* + 1, so that is *m*(*n* + 1) qubits. In the protocol III of ref. 22, the number of the prepared qubits is *mn*(*n* + 1). In ref. 17, Alice need prepare *t* particles, so that is *mt* qubits. In ref. 27, Alice need prepare *t* EPR pairs, so that is 2*mt* qubits.

**Table 1:** Comparison of universality and practicability.

Property	Ref. 7	Ref. 17	I of Ref. 22	III of Ref. 22	Ref. 27	The proposed QSS
(*t*, *n*) or (*n*, *n*)	(*n*, *n*)	(*t*, *n*)	(*n*, *n*)	(*n*, *n*)	(*t*, *n*)	(*t*, *n*)
Level	*d*	2	*d*	*d*	2	*d*
Qubits	*mn*	*mt*	*m*(*n* + 1)	*mn*(*n* + 1)	2*mt*	*mt*

From the Table 1 we can see that refs 17 and 27 and the proposed QSS are (*t*, *n*) schemes, and the three other QSSs are (*n*, *n*) schemes. Ref. 7, the protocol I and III of ref. 22, and the proposed QSS are *d*-level schemes, and the two other QSSs are 2-level schemes. The proposed QSS scheme has not only the merits of (*t*, *n*) scheme but also the merits of *d*-level scheme. It should has better flexibility, universality and practicability than the five other QSS schemes. Moreover, the proposed QSS prepares the same number of the particles as ref. 17, and both schemes can save more resources on the prepared particles than the four other similar schemes.

#### Computation Cost

Ref. 7 does not show how to prepare an *n*-particle entangled state, and ref. 27 also does not describe how to prepare an ordered sequence of *t* EPR pairs. Therefore, in order to make a simplified comparison, we do not consider computation cost of preparing the particles in the protocol I and III of ref. 22 and the proposed QSS scheme. Refs 17 and 27 and the proposed QSS describe the generation process of the shares, however, refs 7 and 22 make no reference to it. Also we do not consider computation cost of the generation process of the shares. In refs 17 and 27, each particle is 2-level. Differently, in refs 7 and 22 and the proposed QSS, each particle is *d*-level. To be convenient for comparison, the particle dimension *d* is to be set to 2, thus $m=\lceil {\mathrm{log}}_{2}\,d\rceil =1$.

The computation costs of the six similar schemes are shown in Table 2. In ref. 7, each participant first performs *QFT* on his particle |*k*〉_*r*_ $(r=1,3,\ldots ,n)$, and then applies ${U}_{{s}_{r}\mathrm{,0}}|k\rangle$ to the particle *QFT*|*k*〉_*r*_, further measures the transformed particle in his lab. The total computation cost is $nQFT+n{U}_{{s}_{r}\mathrm{,0}}+nM$.

**Table 2:** Comparison of computation costs.

Operations of the entities	Ref. 7	Ref. 17	I of Ref. 22	III of Ref. 22	Ref. 27	The proposed QSS
*QFT*	*n*		1	*n* + 1		1
*U* operation	$n{U}_{{s}_{r}\mathrm{,0}}$	*t*(*t* + 1) *U*(*θ*)	(*n* − 1) *U* ^*j*^	(*n* ^2^ − 1) *U* ^*j*^	*t*(2*t* − 1) *U*_*i*,*j*_	$t{U}_{\mathrm{0,}{s}_{r}}$
*QFT* ^−1^			1	*n* + 1		1
Measure operation (*M*)	*n*		2	2(*n* + 1)	*t*	1
Hash operation (*H*)						2*H*

In ref. 17, the dealer Alice performs *U*(*θ*_*a*_) on every particle of the sequence ${\psi }_{0}$, and then sends the transformed sequence to the participant *Bob*_*i*_. For $r=1,2,\ldots ,t$, the participant *Bob*_*r*_ applies *U*(*θ*_*r*_) to the particle sequence ${\psi }_{r-1}$ received from $Bo{b}_{r-1}$, and then sends the transformed sequence to subsequent participant $Bo{b}_{r+1}$. The total computation cost is $t(t+\mathrm{1)}\,U(\theta )$.

In the protocol I of ref. 22, the initiator performs *QFT* on the first particle, and sends the second particle (ancillary particle) to next participant. For $r=2,3,\ldots ,n$, each participant *Bob*_*r*_ performs unitary operation *U* ^*j*^ on his particle. Finally, *Bob*_1_ performs *QFT* ^−1^ on his particle, and then measures it to obtain the secret. The total computation cost of the protocol I is $1QFT+(n-\mathrm{1)}\,{U}^{j}+1QF{T}^{-1}+2M$. To resist collusion attack, the protocol I is upgraded to the protocol III. For $r=1,2,\ldots ,n$, each participant splits his share into *n* pieces, and then calls the protocol I to compute each *y*_*r*_. Finally, one of the participants calls protocol I to compute the summation of all *y*_*r*_. The total computation cost of the protocol III is $(n+1)\,\mathrm{(1}QFT+(n-\mathrm{1)}{U}^{j}+1QF{T}^{-1}+2M)$.

In ref. 27, the dealer first sends the *Y*′ sequence to *Bob*_1_. For $r=1,2,\ldots ,t-1$, *Bob*_*r*_ performs ${U}_{i,j}(i,j\in \{0,1\})$ on each particle of the *Y*′ sequence received from $Bo{b}_{r-1}$. $Bo{b}_{t}$ performs final operation $U={U}_{{B}_{1}}{U}_{{B}_{2}}\cdots {U}_{{B}_{t}}$ on each particle of the transformed *Y*′ sequence received from $Bo{b}_{t-1}$. The total computation cost is $t\mathrm{(2}t-\mathrm{1)}{U}_{i,j}+tM$.

In the proposed QSS, after $Bo{b}_{1}$ performs *QFT* on the first particle |*k*〉_1_, each participant *Bob*_*r*_ $(r=1,2,\ldots ,t)$ applies ${U}_{0,{s}_{r}}={\sum }_{k=0}^{d-1}\,{\omega }^{{s}_{r}\cdot k}{|k\rangle }_{rr}\langle k|$ to his particle |*k*〉_*r*_. Finally, *Bob*_1_ performs *QFT* ^−1^ on his own particle, and then measures it to obtain the secret. The total computation cost is $1QFT+t{U}_{\mathrm{0,}{s}_{r}}+1QF{T}^{-1}+1M+2H$.

The computation cost of Hash operations 2*H* has slight impact on the total cost of the proposed QSS. For a single qubit, *QFT* is a Hadamard gate operation, which is taken as a unitary operation. We assume that the computation costs for each unitary operation in Table 2 are roughly the same. If 2 ≤ *t* = *n*, the computation cost of the proposed QSS and that of the protocol I of ref. 22 are roughly the same, and both are lower than that of the four other schemes. If 2 < *t* < *n* − 1, the computation cost of the proposed QSS is lowest in the six similar schemes.

#### Communication Cost

For the six similar schemes, we assume that the number of the decoy particles is *l*, and the number of the message particles is the same as that of the prepared single-particles or EPR pairs. In ref. 17, the transmission route of the quantum sequence is determined as: *Alice* → *Bob*_*i*_ → *Bob*_1_ → $\cdots$ → *Bob*_*t*_. The total number of the transmitted particles is the sum of the message particles and the decoy particles, as shown in Table 3, which is $(t+l)\,(t+\mathrm{1)}$. In ref. 27, the transmission route of the *Y*′ sequence is determined as: *Alice* → *Bob*_1_ → $\cdots$ → *Bob*_*t*_, and that of the *X*′ sequence is determined as: *Alice* → *Bob*_*t*_. The total number of the transmitted particles is also $(t+l)\,(t+\mathrm{1)}$.

**Table 3:** Comparison of communication costs.

Transmitted particles	Ref. 7	Ref. 17	I of Ref. 22	III of Ref. 22	Ref. 27	The proposed QSS
message particles(or initial particles)	*n* − 1	*t*(*t* + 1)			*t*(*t* + 1)	*t* − 1
decoy particles (or ancillary particles)		*l*(*t* + 1)	*n*	*nn*	*l*(*t* + 1)	

In the protocol I of ref. 22, the ancillary particle is transmitted from one participant to another, and its transmission route is determined as: *Initiator* → *Bob*_2_ → $\cdots$ → *Bob*_*n*_ → *Initiator*. The total number of the transmitted particles is *n*. In the protocol III of ref. 22, for $r=1,2,\ldots ,n$, each participant splits his share into *n* pieces, and every *n* pieces need one ancillary particle to compute *y*_*r*_. The total number of the transmitted particles is *nn*.

In the proposed QSS and ref. 7, the decoy particles are not inserted into the message particles, and the transformed message particles are not transmitted in the quantum channel from one participant to another. The communication cost only is dominated by the distribution of the initial particles from the dealer (or the reconstructor) to every participant. The number of the transmitted particles of the proposed QSS is *t* − 1, and that of ref. 7 is *n* − 1. If *t* = *n*, the communication cost of the proposed QSS, that of ref. 7 and that of the protocol I of ref. 22 are roughly the same. If *t* < *n*, the communication cost of the proposed QSS is the lowest in the six similar schemes.

## Discussion

Some existing QSS schemes cannot resist collusion attack of the participants, and the unqualified subsets set of participants can obtain some information about the secret. To resist collusion attack, ref. 22 upgraded the protocol I to the protocol III. With the enhancement of the security, the computation cost of the protocol III flies to (*n* + 1) times. In this paper, we present a (*t*, *n*) threshold *d*-level QSS scheme. Security analysis shows that our scheme can also resist collusion attack. Furthermore, if 2 < *t* < *n* − 1, our scheme has lower computation and communication cost than other similar schemes including the protocol I of ref. 22. Our scheme is feasible and practical with the present technologies, because it employs quantum *CNOT*, *QFT*, and Pauli operator ${U}_{0,{s}_{r}}$ as main transformation operations, which have been used widely in quantum field.
